# Personalized three-year survival prediction and prognosis forecast by interpretable machine learning for pancreatic cancer patients: a population-based study and an external validation

**DOI:** 10.3389/fonc.2024.1488118

**Published:** 2024-10-21

**Authors:** Buwei Teng, Xiaofeng Zhang, Mingshu Ge, Miao Miao, Wei Li, Jun Ma

**Affiliations:** ^1^ Department of Hepatobiliary Surgery, The Affiliated Lianyungang Hospital of Xuzhou Medical University/The First People’s Hospital of Lianyungang, Lianyungang, China; ^2^ Department of Imaging, The Affiliated Huai’an Hospital of Xuzhou Medical University and the Second People’s Hospital of Huai’an, Huai’an, China

**Keywords:** machine learning, pancreatic cancer, three-year survival, prognosis prediction, SEER

## Abstract

**Purpose:**

The overall survival of patients with pancreatic cancer is extremely low. We aimed to establish machine learning (ML) based model to accurately predict three-year survival and prognosis of pancreatic cancer patients.

**Methods:**

We analyzed pancreatic cancer patients from the Surveillance, Epidemiology, and End Results (SEER) database between 2000 and 2021. Univariate and multivariate logistic analysis were employed to select variables. Recursive Feature Elimination (RFE) method based on 6 ML algorithms was utilized in feature selection. To construct predictive model, 13 ML algorithms were evaluated by area under the curve (AUC), area under precision-recall curve (PRAUC), accuracy, sensitivity, specificity, precision, cross-entropy, Brier scores and Balanced Accuracy (bacc) and F Beta Score (fbeta). An optimal ML model was constructed to predict three-year survival, and the predictive results were explained by SHapley Additive exPlanations (SHAP) framework. Meanwhile, 101 ML algorithm combinations were developed to select the best model with highest C-index to predict prognosis of pancreatic cancer patients.

**Results:**

A total of 20,064 pancreatic cancer patients from SEER database was consecutively enrolled. We utilized eight clinical variables to establish prediction model for three-year survival. CatBoost model was selected as the best prediction model, and AUC was 0.932 [0.924, 0.939], 0.899 [0.873, 0.934] and 0.826 [0.735, 0.919] in training, internal test and external test sets, with 0.839 [0.831, 0.847] accuracy, 0.872 [0.858, 0.887] sensitivity, 0.803 [0.784, 0.825] specificity and 0.832 [0.821, 0.853] precision. Surgery type had the greatest effects on three-year survival according to SHAP results. For prognosis prediction, “RSF+GBM” algorithm was the best prognostic model with C-index of 0.774, 0.722 and 0.674 in training, internal test and external test sets.

**Conclusions:**

Our ML models demonstrate excellent accuracy and reliability, offering more precise personalized prognostic prediction to pancreatic cancer patients.

## Introduction

Pancreatic cancer is a highly lethal disease with a dismal prognosis, and the 5-year survival rate is merely 9% (1). Only 1% of patients survive for 3 years or more after diagnosis of metastatic pancreatic cancer, while the incidence continues to climb steadily. Surgical resection is the only potential curative treatment, yet only a small proportion of pancreatic cancer patients are eligible for surgery at the time of initial diagnosis (2). This is largely because pancreatic cancer often lacks symptoms in its early stages, leading to most cases being diagnosed at an advanced stage (3). While some individuals may detect the disease through routine physical examinations and undergo early surgery, many patients still experience relapse and ultimately succumb to the disease (4). The treatment of pancreatic cancer mainly includes surgical resection, radiotherapy, chemotherapy and targeted therapy, but the overall efficacy is limited due to its high aggressiveness and the norm of late detection. Novel drugs targeting the KRAS gene, such as sotorasib and adagrasib, have demonstrated efficacy and tolerability in treating solid tumors, including pancreatic cancer, in clinical trials (5). Consequently, it is critical to promptly and early identify pancreatic cancer patients at high risk to optimize their treatment and improve prognosis. And exploring the prognostic risk factors for pancreatic cancer patients is crucial to assess their survival prospects.

Several biomarkers for prognosis prediction in pancreatic cancer have been identified in recent years, including CA19-9, circulating tumor DNA (ctDNA), microRNAs (miRNAs), and tumor mutational burden (TMB) (6). However, CA19-9 is not specific to pancreatic cancer and can be elevated in other conditions such as cholangitis, leading to false positives. Meanwhile, ctDNA analysis is limited by the low abundance of tumor DNA in the bloodstream, particularly in early-stage cancers, which may result in false negatives. And the clinical application of miRNAs is still in the early stages, and their stability in circulation poses challenges for reliable detection (7). Furthermore, TMB’s predictive value is still under investigation, and its utility may vary depending on the genetic landscape of the tumor and the therapeutic context (8). Recently, nomogram based on Cox model has been widely utilized in cancer prognosis prediction, but its sensitivity and specificity may be insufficient, calling an urgent need for predicting prognosis more accurately and specifically. Machine learning (ML) approach, a subset of artificial intelligence, has become increasingly popular due to its ability to handle complex, non-linear relationships, particularly effective with vast datasets and loosely structured information (9). With the advent of big data analytics and ML, new approaches for screening risk factors affecting prognosis have become feasible. Several predictive models leveraging these technologies have shown excellent performance and are increasingly being integrated into clinical settings (10, 11), while there is no ML-based sophisticated model to predict prognosis in pancreatic cancer so far, necessitating development and validation of a novel ML model.

The Surveillance, Epidemiology, and End Results (SEER) database (https://seer.cancer.gov/) is particularly valuable in this context, which encompasses a wide range of patient data, offering comprehensive clinicopathological statistics and follow-up information. This rich, real-world database is an ideal resource for developing and testing ML models in the medical field. However, it appears that there is still a gap in research specifically focused on developing models for three-year survival prediction and prognosis forecast of pancreatic cancer patients. Our study was committed to firstly developing and validating predictive and prognostic models utilizing multiple ML algorithms. This approach leverages extensive population data and the capabilities of ML, which is competent in providing personalized predictive tools that assist clinicians in effectively assessing the risk and prognosis of pancreatic cancer patients.

## Materials and methods

### Data source and characteristics

Clinicopathological data of patients with site recode ICD-O-3/WHO 2008 “pancreas” and AYA site recode 2020 Revision “9.3.9.2 Pancreas – adenocarcinoma” between 2000 and 2021 was retrieved from the SEER database. Additionally, clinicopathological information of pancreatic The First People’s Hospital of Lianyungang (2015–2024) was retrospectively collected through electronic medical record system. The study was conducted according to the guidelines of the Declaration of Helsinki and was approved by the Ethics Committee of The First People’s Hospital of Lianyungang (protocol code: KY-20210910004, approved on 2021-09-10). Informed consent was obtained from all subjects involved in this study. Inclusion criteria comprised individuals with ICD-O-3/WHO 2008 “pancreas” and AYA site recode 2020 Revision “9.3.9.2 Pancreas – adenocarcinoma” which are older than 18 years old. Exclusion criteria comprised patients lacking follow-up information of survival months and death cause, not diagnosed with positive histology, no surgery information, not first malignant tumor, without TNM stage or grade details. In SEER database, metastasis is characterized by spreading to distant organs during the initial cancer diagnosis. And we define the outcome of predictive model as three-year survival, indicating that patients are still alive at the timepoint of 36 months follow-up. The positive outcome was death of patient in three-year follow-up.

Extracted data were gathered on demographic data (age, gender, race, marital status household location and income), cancer characteristics (pathological grade, summary stage, TNM stage, tumor size, tumor primary location, pathology, metastasis information), therapeutic information (surgery, lymph node surgery, positive lymph node, radiotherapy, chemotherapy) and follow-up information (overall and cancer-specific survival status, survival months). Two continuous variables, age and tumor size, were divided into categorical variables. The age was split into five groups: “<50”, “50-59”, “60-69”, “70-79” and “>=80”. The tumor size was split into “<2cm”, “2-3.9cm”, “4-5.9cm”, “6-7.9cm”, “>8cm” and “Unknown”. “Metastasis” was defined as “yes” with metastasis either in brain, bone, liver, lung, and distant lymph nodes, as well as tumor categorized as M1 stage. The missing rate for each categorical variable is calculated and reported. For those classified data that is unknown, we classify its missing value into the “unknown” category. This processing ensures data integrity and avoids information loss due to missing data. We determined the minimum sample size needed for an external validation cohort by formula of Riley et al. (12).

### Establishment and validation of predictive model for three-year survival

In the preliminary analysis, variables with P < 0.05 in the univariate and multivariate logistic analysis in the training set were included for the feature selection process. Subsequently, we employed Recursive Feature Elimination (RFE) method based on 6 ML algorithms, involving categorical boosting (CatBoost), random forest (RF), support vector machine (SVM), extreme gradient boosting (XGB), decision tree (DT) and gradient boosting machine (GBM), combined with 5-fold cross-validation, to sift through the clinical features. RFE works by building a model and identifying the most significant features in feature selection phase. This selection process is then iteratively repeated on the subset of remaining features until all features have been evaluated and ranked (13). Then Robust rank aggregation (RRA) algorithm was utilized to integrate the rank of variable importance from six ML algorithms utilized in RFE method to obtain a comprehensive ranking of all variables (14). We set random seed as “2024” in our analysis. In model development phase, we applied 13 ML algorithms, including CatBoost, RF, SVM, XGB, DT, GBM, k-nearest neighbor (KNN), logistic regression (LR), naive bayes classifier (NBC), linear discriminant analysis (LDA), quadratic discriminant analysis (QDA), neural network (NNET) and generalized linear model (GLM) to predict three-year survival via “mlr3” R package (15). This approach allows us to compare the performance of various models and select the best predictive model. To tackle the issue of class imbalance, which could significantly skew performance metrics, we implemented the Synthetic Minority Over-sampling Technique (SMOTE) for training our model (16). We further refined our approach by employing nested resampling, which involved a two-tiered k-fold cross-validation process: one for optimizing model hyperparameters and another nested within it for model selection. Meanwhile, we utilized a 1000-evaluation random search across a 5-fold cross-validation framework, repeated five times for each model. Subsequently, area under the curve (AUC), area under precision-recall curve (PRAUC), accuracy, sensitivity, specificity, precision, cross-entropy, Brier scores and Balanced Accuracy (bacc) and F Beta Score (fbeta) were calculated to select the best ML model. Internal validation was carried out through 5-fold cross-validation. Precision-recall curve (PRC) was employed to evaluate the performance of classification models in handling imbalanced datasets. Calibration curve was utilized to appraise model’s discriminative ability, and decision curve analysis (DCA) was applied to verify the clinic benefit of ML model via “runway” R package (https://github.com/ML4LHS/runway/). We set the selection criteria of our best model: highest AUC, highest PRAUC, and lowest Brier score, while also ensuring a good calibration curve, as well as outperforming balanced accuracy and F Beta Score. To quantify the impact of each variable, we calculated its mean contribution to the AUC as a percentage relative to the full model via “DALEX” R package (17). SHapley Additive exPlanations (SHAP) value were used to explain the best model predictions and to interpret the black-box ML model via “shapviz” R package (https://github.com/ModelOriented/shapviz) (18).

### Prognostic model based on integrative machine learning algorithms

Univariate and multivariate cox analysis were employed to define clinical variables with significant prognosis value in overall survival (OS). We integrated 10 ML algorithms involving random survival forest (RSF), elastic network (Enet), Lasso, Ridge, stepwise Cox, CoxBoost, partial least squares regression for Cox (plsRcox), supervised principal components (SuperPC), GBM and survival support vector machine (survival-SVM) to predict prognosis (in terms of OS) of pancreatic cancer patients. Altogether 101 prognostic ML algorithm combinations were trained in the training cohort, to develop the prognostic ML model according to the leave-one-out cross-validation (LOOCV) framework. Models with <3 clinical variable were removed. Subsequently, the concordance index (C-index) of every ML combination in training, testing and external validation cohorts was obtained (19). The top five ML combinations yielding the highest average C-index across three cohorts were selected for model evaluation via k-fold cross-validation, to mitigate overfitting and ensure the robustness and generalizability of model. Logarithmic loss, recall and decision calibration were utilized to select the best prognostic ML combination via “mlr3proba” R package (20). We incorporated variables from various feature selection patterns to compute risk scores using a linear combination function for each prognostic ML combination. The median risk score from the training cohort was chosen as the threshold to categorize patients in training, testing and external validation cohorts into high or low-risk groups. We utilized the Kaplan-Meier (KM) survival analysis and the log-rank test on these groups, using the “survival” and “survminer” R packages. AUC, time-dependent receiver operating characteristic (ROC) curves, calibration curves and DCA were employed to evaluate the precision, discrimination and clinical benefit of the model.

## Results

### Demographic composition and clinical baseline information

**Figure 1:**
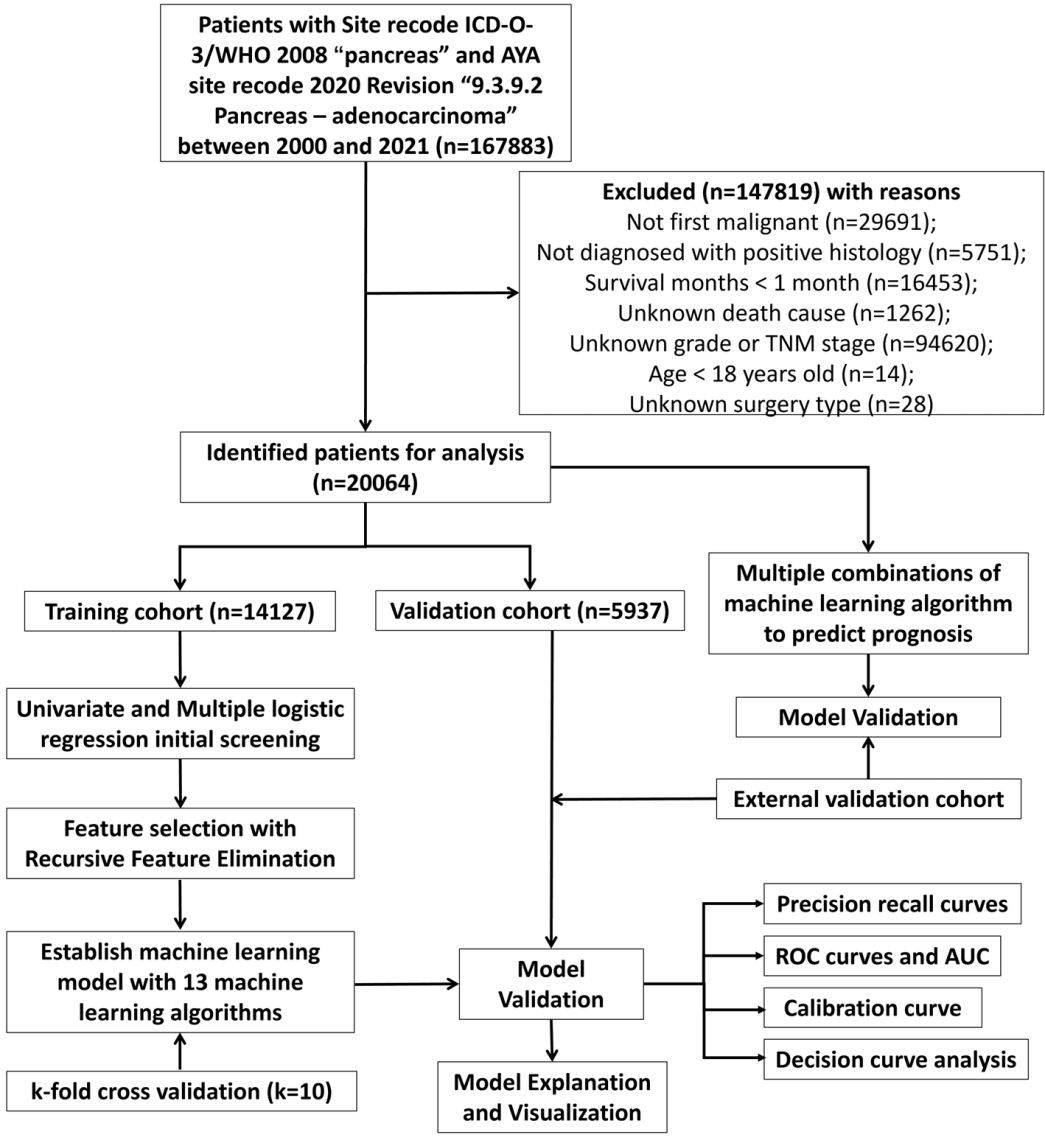
The workflow diagram for study design and patient screening.

In the predictive model for three-year survival, a total of 20064 pancreatic cancer patients from SEER database and 103 patients from The First People’s Hospital of Lianyungang were included. We divided patients from SEER database randomly into training and internal validation sets in a 7:3 ratio, respectively. And pancreatic cancer patients from The First People’s Hospital of Lianyungang were assigned as the external validation set. In the trainset from SEER database, 2579 cases (18.3%) were alive at three-year follow-up, while 11548 cases (81.7%) did not. Detailed clinical information regarding the training and validation sets to predict three-year survival can be found in **Table 1**. For the outcomes (in terms of OS) of prognostic model, 13157 cases (93.1%) were dead at the time of follow-up, while 970 cases (6.87%) were alive (**Table 1**). In the training, internal validation and external validation sets, the median follow-up time was 12.0 [5.00;26.0], 12.0 [5.00;27.0] and 16.0 [6.00;30.5] (**Table 1**). The specific selection process of patients from SEER database is shown in **Figure 1**.

**Table 1:** Clinicopathological characteristics of patients with pancreatic cancer in the training, internal validation and external validation cohorts.

	Training Cohort N=14127	Validation Cohort N=5937	External Validation Cohort N=103	p.overall
Sex				0.997
Male				
Female	7162 (50.7%)	3013 (50.7%)	52 (50.5%)	
Age				0.046
<50	1048 (7.42%)	419 (7.06%)	11 (10.7%)	
50-59	2928 (20.7%)	1223 (20.6%)	21 (20.4%)	
60-69	4567 (32.3%)	1963 (33.1%)	17 (16.5%)	
70-79	3936 (27.9%)	1654 (27.9%)	38 (36.9%)	
>=80	1648 (11.7%)	678 (11.4%)	16 (15.5%)	
Race				<0.001
White	11343 (80.3%)	4772 (80.4%)	0 (0.00%)	
Black	1558 (11.0%)	641 (10.8%)	0 (0.00%)	
Other	1226 (8.68%)	524 (8.83%)	103 (100%)	
Marital_Status				0.378
Married	8571 (60.7%)	3614 (60.9%)	69 (67.0%)	
Unmarried	1782 (12.6%)	720 (12.1%)	14 (13.6%)	
Widowed or divorced	3365 (23.8%)	1418 (23.9%)	20 (19.4%)	
Unknown	409 (2.90%)	185 (3.12%)	0 (0.00%)	
Year_of_Diagnosis				<0.001
2000-2010	7649 (54.1%)	3221 (54.3%)	78 (75.7%)	
2011-2020	6478 (45.9%)	2716 (45.7%)	25 (24.3%)	
Household_Location				0.684
Rural	1587 (11.2%)	656 (11.0%)	9 (8.74%)	
Urban	12540 (88.8%)	5281 (89.0%)	94 (91.3%)	
Household_Income				<0.001
<$70,000	4765 (33.7%)	1974 (33.2%)	9 (8.74%)	
>=$70,000	9362 (66.3%)	3963 (66.8%)	94 (91.3%)	
Tumor_Primary_Site				0.519
Pancreas Head	9330 (66.0%)	3924 (66.1%)	63 (61.2%)	
Pancreas Body or Tail	2910 (20.6%)	1259 (21.2%)	24 (23.3%)	
Other	1887 (13.4%)	754 (12.7%)	16 (15.5%)	
Histology				0.633
Adenomas and adenocarcinomas	9285 (65.7%)	3940 (66.4%)	73 (70.9%)	
Ductal and lobular neoplasms	3769 (26.7%)	1586 (26.7%)	23 (22.3%)	
Cystic, mucinous and serous neoplasms	854 (6.05%)	329 (5.54%)	6 (5.83%)	
Other	219 (1.55%)	82 (1.38%)	1 (0.97%)	
Grade				0.629
Well differentiated I	1673 (11.8%)	709 (11.9%)	13 (12.6%)	
Moderately differentiated II	6606 (46.8%)	2783 (46.9%)	48 (46.6%)	
Poorly differentiated III	5645 (40.0%)	2376 (40.0%)	39 (37.9%)	
Undifferentiated anaplastic IV	203 (1.44%)	69 (1.16%)	3 (2.91%)	
Summary_Stage				0.186
Localized	1341 (9.49%)	533 (8.98%)	16 (15.5%)	
Regional	8635 (61.1%)	3636 (61.2%)	57 (55.3%)	
Distant	4151 (29.4%)	1768 (29.8%)	30 (29.1%)	
AJCC_Stage				0.360
I	1341 (9.49%)	533 (8.98%)	16 (15.5%)	
II	8000 (56.6%)	3392 (57.1%)	54 (52.4%)	
III	1448 (10.2%)	590 (9.94%)	10 (9.71%)	
IV	3338 (23.6%)	1422 (24.0%)	23 (22.3%)	
T_Stage				0.459
T1	633 (4.48%)	284 (4.78%)	7 (6.80%)	
T2	2352 (16.6%)	964 (16.2%)	22 (21.4%)	
T3	8764 (62.0%)	3718 (62.6%)	56 (54.4%)	
T4	2378 (16.8%)	971 (16.4%)	18 (17.5%)	
N_Stage				0.758
N0	6526 (46.2%)	2776 (46.8%)	47 (45.6%)	
N1	7601 (53.8%)	3161 (53.2%)	56 (54.4%)	
M_Stage				0.839
M0	10789 (76.4%)	4515 (76.0%)	80 (77.7%)	
M1	3338 (23.6%)	1422 (24.0%)	23 (22.3%)	
Tumor_Size				0.297
<2cm	1084 (7.67%)	472 (7.95%)	11 (10.7%)	
2-3.9cm	6743 (47.7%)	2798 (47.1%)	47 (45.6%)	
4-5.9cm	3896 (27.6%)	1657 (27.9%)	29 (28.2%)	
6-7.9cm	1050 (7.43%)	427 (7.19%)	10 (9.71%)	
>8cm	470 (3.33%)	211 (3.55%)	6 (5.83%)	
Unknown	884 (6.26%)	372 (6.27%)	0 (0.00%)	
Surgery_Type				0.180
No Surgery	5646 (40.0%)	2408 (40.6%)	41 (39.8%)	
Local or partial pancreatectomy	6947 (49.2%)	2829 (47.7%)	48 (46.6%)	
Total pancreatectomy	1534 (10.9%)	700 (11.8%)	14 (13.6%)	
Lymph_Nodes_Surgery				0.743
No or biopsy only	5696 (40.3%)	2441 (41.1%)	42 (40.8%)	
1-3 regional lymph nodes removed	787 (5.57%)	342 (5.76%)	7 (6.80%)	
4 or more regional lymph nodes removed	7644 (54.1%)	3154 (53.1%)	54 (52.4%)	
Regional_Lymph_Nodes				0.676
No nodes were examined	5302 (37.5%)	2277 (38.4%)	38 (36.9%)	
Negative	3059 (21.7%)	1267 (21.3%)	25 (24.3%)	
Positive	5729 (40.6%)	2384 (40.2%)	40 (38.8%)	
Unknown	37 (0.26%)	9 (0.15%)	0 (0.00%)	
Chemotherapy				0.911
None/Unknown	4666 (33.0%)	1945 (32.8%)	35 (34.0%)	
Yes	9461 (67.0%)	3992 (67.2%)	68 (66.0%)	
Radiation				0.328
None/Unknown	9995 (70.8%)	4174 (70.3%)	79 (76.7%)	
Yes	4132 (29.2%)	1763 (29.7%)	24 (23.3%)	
Metastasis				0.940
No	5859 (41.5%)	2465 (41.5%)	41 (39.8%)	
Yes	8268 (58.5%)	3472 (58.5%)	62 (60.2%)	
Bone_Metastasis				0.804
No	7530 (53.3%)	3180 (53.6%)	54 (52.4%)	
Yes	97 (0.69%)	47 (0.79%)	0 (0.00%)	
Unknown	6500 (46.0%)	2710 (45.6%)	49 (47.6%)	
Brain_Metastasis				0.485
No	7620 (53.9%)	3227 (54.4%)	54 (52.4%)	
Yes	5 (0.04%)	0 (0.00%)	0 (0.00%)	
Unknown	6502 (46.0%)	2710 (45.6%)	49 (47.6%)	
Liver_Metastasis				0.492
No	6381 (45.2%)	2667 (44.9%)	48 (46.6%)	
Yes	1264 (8.95%)	569 (9.58%)	6 (5.83%)	
Unknown	6482 (45.9%)	2701 (45.5%)	49 (47.6%)	
Lung_Metastasis				0.778
No	7288 (51.6%)	3073 (51.8%)	51 (49.5%)	
Yes	329 (2.33%)	154 (2.59%)	3 (2.91%)	
Unknown	6510 (46.1%)	2710 (45.6%)	49 (47.6%)	
Survival_Months	12.0 [5.00;26.0]	12.0 [5.00;27.0]	16.0 [6.00;30.5]	0.605
Vital_Status				0.848
Alive	970 (6.87%)	421 (7.09%)	7 (6.80%)	
Dead	13157 (93.1%)	5516 (92.9%)	96 (93.2%)	
Cancer_Specific_Death				0.440
Not cancer specific death	1823 (12.9%)	799 (13.5%)	11 (10.7%)	
Dead due to bladder cancer	12304 (87.1%)	5138 (86.5%)	92 (89.3%)	
Other_Cause_Death				0.430
Not other cause death	13274 (94.0%)	5559 (93.6%)	99 (96.1%)	
Dead due to other cause	853 (6.04%)	378 (6.37%)	4 (3.88%)	
Three_Year_Survival				0.911
Alive	2579 (18.3%)	1097 (18.5%)	18 (17.5%)	
Dead	11548 (81.7%)	4840 (81.5%)	85 (82.5%)	

### Feature selection for the predictive model

**Figure 2:**
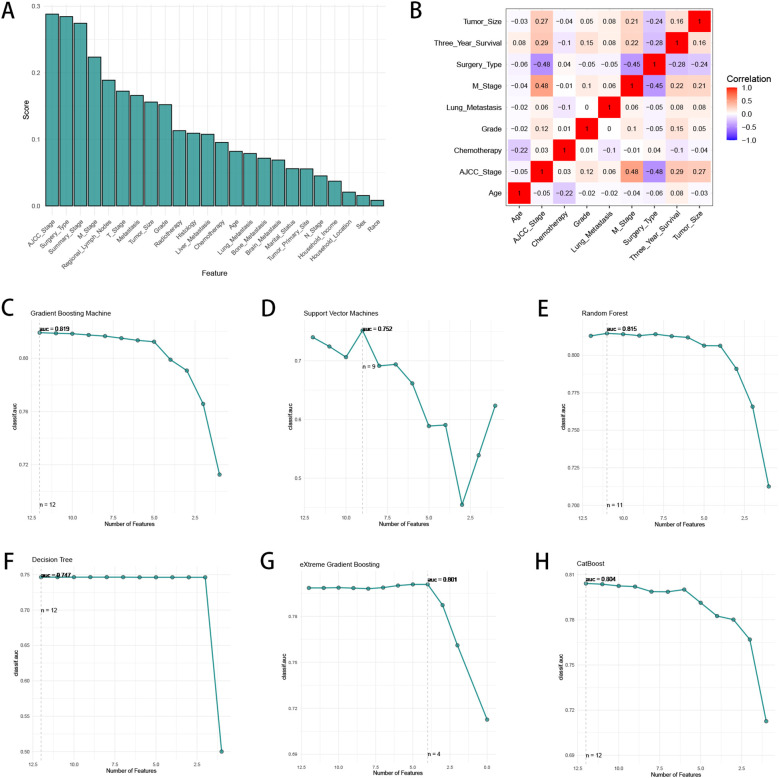
The process of feature selection. **(A)** The correlation coefficients of the baseline characteristics with three-year survival. **(B)** The heatmap of Spearman’s correlation analysis of the clinic variables with three-year survival. The correlation index ranges from -1.0 to 1.0, with a brighter color indicating a stronger correlation. **(C–H)** Feature selection process with Recursive Feature Elimination (RFE) method based on six ML algorithms (GBM, SVM, RF, DT, XGB and CatBoost).

We utilized “autoplot” function in “mlr3” R package to visualized the correlation coefficients of the baseline characteristics with three-year survival, which revealed that “AJCC stage” had the most significant correlation with three-year survival (**Figure 2A**). Based on our clinical experiences, we selected 24 variables for the logistic regression analysis (**Table 2**), while the variable with a correlation coefficient > 0.6 was removed. Subsequently, we performed univariate and multivariate logistic regression analysis in the training cohort to find the effective variables to predict three-year survival, which revealed that “Age” (OR 1.67(1.35-2.06)), “Marital_Status” (OR 1.27(1.14-1.42)), “Household_Income” (OR 0.75(0.68-0.83)), “Histology” (OR 0.5(0.43-0.59)), “Grade” (OR 2.4(1.59-3.72)), “Summary_Stage” (OR 3.12(2.27-4.31)), “Tumor_Size” (OR 2.63(2.08-3.34)), “AJCC_Stage” (OR 1.59(1.09-2.35)), “Surgery_Type” (OR 0.15(0.11-0.2)), “Radiotherapy” (OR 0.79(0.72-0.87)), “Chemotherapy” (OR 0.57(0.52-0.64)), “Lung_Metastasis” (OR 0.2(0.06-0.99)), “M_Stage” (OR 1.26(1.07-1.48)) were significantly powerful to predict three-year survival (P < 0.05, **Table 2**). The correlation analysis between the variables and three-year survival showed that “AJCC_stage” is the most influential factor (**Figure 2B**). Due to high correlation between “AJCC_stage” and “Summary_Stage”, we only choose “AJCC_stage” in the following analysis. Afterwards, we utilized Recursive Feature Elimination (RFE) method based on six ML algorithms (GBM, SVM, RF, DT, XGB and CatBoost), combined with 5-fold cross-validation, to sift through the clinic features (**Figures 2C–H**). Feature selection based on RFE found that the optimal selection was according to GBM algorithm, remaining 12 variables, with the highest AUC (0.819, **Figure 2C**). We utilized RRA algorithm to obtain the comprehensive ranking of the clinic variables in six ML algorithms, with the “AJCC_stage” considered most important (**Supplementary Table 1**). We finally select eight variables with frequencies more than 4, which indicates that these variables are important in most of the ML selection process, into the following procedures of model development (**Supplementary Table 1**).

**Table 2:** Univariate and multivariate logistics analysis of pancreatic cancer patients for 3-year survival in the training cohort.

Variable	Univariable logistic analysis		Multivariate logistic analysis	
term	OR (95%CI)	p.value	OR (95%CI)	p.value
Sex: Male	Reference			
Female	0.92 (0.85-1)	0.064	\	\
Age: <50	Reference			
50-59	1.12 (0.95-1.33)	0.185	1.05 (0.89-1.24)	0.568
60-69	1.15 (0.98-1.35)	0.089	1.18 (1.01-1.39)	0.04
70-79	1.51 (1.28-1.79)	<0.001	1.41 (1.19-1.66)	<0.001
>=80	2.46 (1.99-3.05)	<0.001	1.67 (1.35-2.06)	<0.001
Race: White	Reference			
Black	1.15 (1-1.33)	0.047	1.01 (0.88-1.16)	0.898
Other	1.02 (0.87-1.19)	0.841	1.04 (0.9-1.2)	0.604
Marital_Status: Married	Reference			
Unmarried	1.21 (1.06-1.38)	0.006	1.18 (1.03-1.34)	0.015
Widowed or divorced	1.47 (1.32-1.64)	<0.001	1.27 (1.14-1.42)	<0.001
Unknown	1.21 (0.94-1.59)	0.154	1.3 (1.02-1.68)	0.038
Household_Income: <$70,000	Reference			
>=$70,000	0.81 (0.74-0.89)	<0.001	0.75 (0.68-0.83)	<0.001
Household_Location: Rural				
Urban	0.84 (0.73-0.96)	0.014	0.94 (0.81-1.08)	0.386
Tumor_Primary_Site: Pancreas Head	Reference			
Pancreas Body or Tail	1.27 (1.14-1.42)	<0.001	0.91 (0.79-1.05)	0.191
Other	1.51 (1.31-1.73)	<0.001	1.05 (0.91-1.21)	0.488
Histology: Adenomas and adenocarcinomas	Reference			
Ductal and lobular neoplasms	0.55 (0.5-0.6)	<0.001	1.05 (0.96-1.15)	0.3
Cystic, mucinous and serous neoplasms	0.41 (0.35-0.48)	<0.001	0.5 (0.43-0.59)	<0.001
Other	0.92 (0.64-1.34)	0.642	0.86 (0.62-1.2)	0.359
Grade: Well differentiated I	Reference			
Moderately differentiated II	1.56 (1.38-1.76)	<0.001	1.77 (1.57-2)	<0.001
Poorly differentiated III	3.12 (2.73-3.56)	<0.001	2.75 (2.41-3.14)	<0.001
Undifferentiated anaplastic IV	2.97 (1.97-4.68)	<0.001	2.4 (1.59-3.72)	<0.001
Summary_Stage: Localized	Reference			
Regional	2.55 (2.26-2.87)	<0.001	1.74 (1.31-2.3)	<0.001
Distant	15.52 (12.92-18.72)	<0.001	3.12 (2.27-4.31)	<0.001
AJCC_Stage: I	Reference			
II	2.34 (2.07-2.64)	<0.001	1.11 (0.89-1.39)	0.336
III	8.99 (7.19-11.33)	<0.001	1.17 (0.93-1.48)	0.179
IV	24.81 (19.71-31.56)	<0.001	1.59 (1.09-2.35)	0.018
T_Stage: T1	Reference			
T2	3.5 (2.9-4.22)	<0.001	1.11 (0.89-1.38)	0.374
T3	3.33 (2.83-3.93)	<0.001	1.17 (0.92-1.47)	0.196
T4	15.41 (12.14-19.67)	<0.001	1.59 (1.03-2.54)	0.051
N_Stage: N0	Reference			
N1	1.26 (1.16-1.38)	<0.001	1.1 (0.86-1.41)	0.448
M_Stage: M0	Reference			
M1	10.69 (8.7-13.32)	<0.001	1.26 (1.07-1.48)	0.005
Tumor_Size: <2cm	Reference			
2-3.9cm	2.75 (2.4-3.15)	<0.001	1.74 (1.5-2.02)	<0.001
4-5.9cm	4.57 (3.92-5.32)	<0.001	2.19 (1.85-2.59)	<0.001
6-7.9cm	5.8 (4.62-7.32)	<0.001	2.63 (2.08-3.34)	<0.001
>8cm	3.62 (2.77-4.79)	<0.001	1.63 (1.22-2.19)	0.001
Unknown	9.43 (7.13-12.67)	<0.001	1.72 (1.3-2.3)	<0.001
Surgery_Type: No Surgery	Reference			
Local or partial pancreatectomy	0.07 (0.06-0.08)	<0.001	0.15 (0.11-0.2)	<0.001
Total pancreatectomy	0.08 (0.06-0.09)	<0.001	0.15 (0.12-0.21)	<0.001
Lymph_Nodes_Surgery: No or biopsy only	Reference			
1-3 regional lymph nodes removed	0.14 (0.11-0.17)	<0.001	1.09 (0.76-1.54)	0.652
4 or more regional lymph nodes removed	0.09 (0.08-0.11)	<0.001	0.74 (0.53-1.02)	0.067
Regional_Lymph_Nodes: No nodes were examined	Reference			
Negative	0.06 (0.05-0.07)	<0.001	0.75 (0.51-1.12)	0.158
Positive	0.14 (0.12-0.17)	<0.001	1.28 (0.88-1.86)	0.196
Unknown	0.19 (0.09-0.52)	<0.001	0.53 (0.21-1.54)	0.205
Chemotherapy: None/Unknown	Reference			
Yes	0.57 (0.51-0.63)	<0.001	0.57 (0.52-0.64)	<0.001
Radiotherapy: None/Unknown	Reference			
Yes	0.55 (0.5-0.6)	<0.001	0.79 (0.72-0.87)	<0.001
Metastasis: No	Reference			
Yes	2.37 (2.17-2.58)	<0.001	0.9 (0.6-1.4)	0.633
Bone_Metastasis: No	Reference			
Yes	12.58 (3.98-76.41)	<0.001	2.29 (0.68-14.29)	0.262
Unknown	1.46 (1.34-1.59)	<0.001	0.83 (0.05-21.63)	0.919
Brain_Metastasis: No	Reference			
Yes	2.92 (0-NA)	0.908	1766.26 (0-NA)	0.958
Unknown	1.44 (1.32-1.57)	<0.001	0.96 (0.06-25.1)	0.984
Liver_Metastasis: No	Reference			
Yes	10 (7.34-14.05)	<0.001	1.03 (0.58-1.79)	0.930
Unknown	1.75 (1.6-1.91)	<0.001	4.39 (0.42-24.26)	0.150
Lung_Metastasis: No	Reference			
Yes	9.75 (5.33-20.47)	<0.001	1.06 (0.51-2.44)	0.877
Unknown	1.51 (1.38-1.64)	<0.001	0.2 (0.06-0.99)	0.023

### Development and validation of predictive model for three-year survival

**Figure 3:**
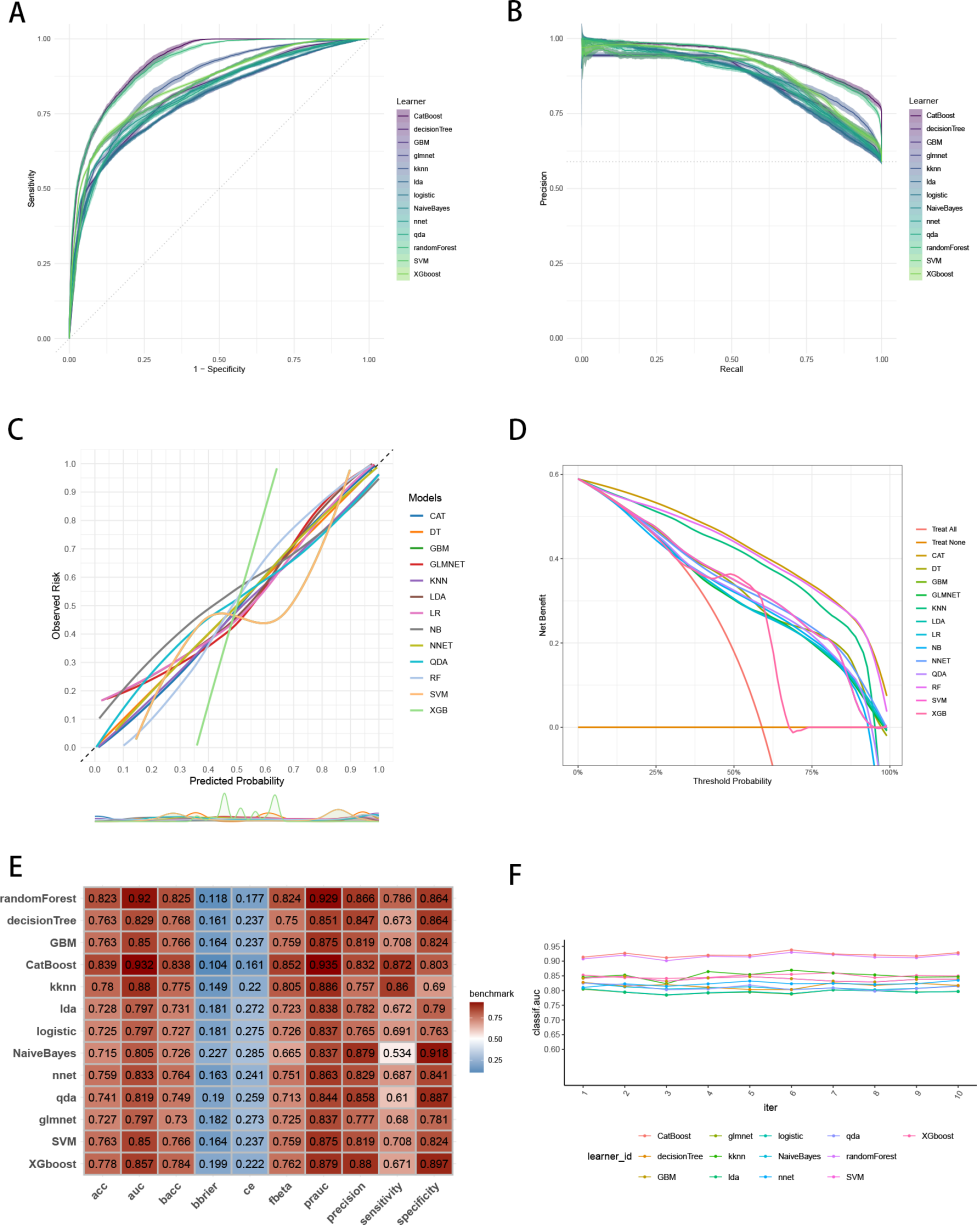
Establishment and evaluation of the ML models in the training set. **(A)** ROC curves of different ML models in the training set. **(B)** PR curves of different ML models in the training set. **(C)** Calibration curves of different ML models in the training set. **(D)** DCA curves of different ML models in the training set. **(E)** The performance of 13 ML models in terms of AUC, PRAUC, accuracy, sensitivity, specificity, precision, cross-entropy, Brier scores and Balanced Accuracy (bacc) and F Beta Score (fbeta) in the training set. **(F)** Ten-fold cross-validation results of different ML models in the training set. ML, machine learning; CAT, categorical boosting; LR, logistic regression; DT, decision tree; RF, random forest; XGB, extreme gradient boosting; GBM, gradient boosting machine; NB, Naive Bayes; LDA, linear discriminant analysis; QDA, quadratic discriminant analysis; NNET, neural network; GLMNET, generalized linear models with elastic net regularization; SVM, support vector machine; KNN, k-nearest neighbor.

**Figure 4:**
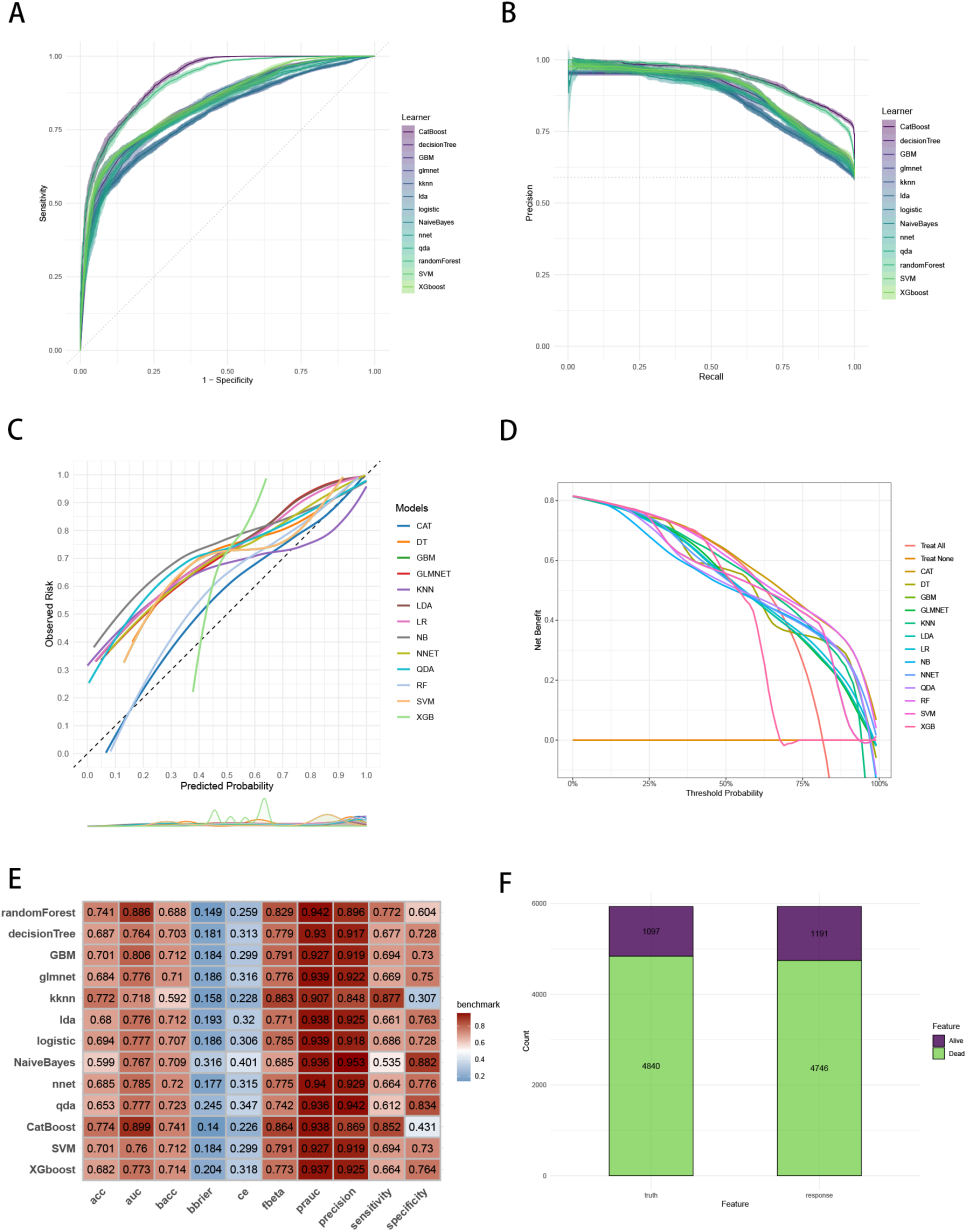
Evaluation of the ML models in the internal validation set. **(A)** ROC curves of different ML models in the internal validation set. **(B)** PR curves of different ML models in the internal validation set. **(C)** Calibration curves of different ML models in the internal validation set. **(D)** DCA curves of different ML models in the internal validation set. **(E)** The performance of 13 ML models in terms of AUC, PRAUC, accuracy, sensitivity, specificity, precision, cross-entropy, Brier scores and Balanced Accuracy (bacc) and F Beta Score (fbeta) in the internal validation set. **(F)** Confusion matrix of the best ML model in the internal validation set. ML, machine learning; CAT, categorical boosting; LR, logistic regression; DT, decision tree; RF, random forest; XGB, extreme gradient boosting; GBM, gradient boosting machine; NB, Naive Bayes; LDA, linear discriminant analysis; QDA, quadratic discriminant analysis; NNET, neural network; GLMNET, generalized linear models with elastic net regularization; SVM, support vector machine; KNN, k-nearest neighbor.

**Figure 5:**
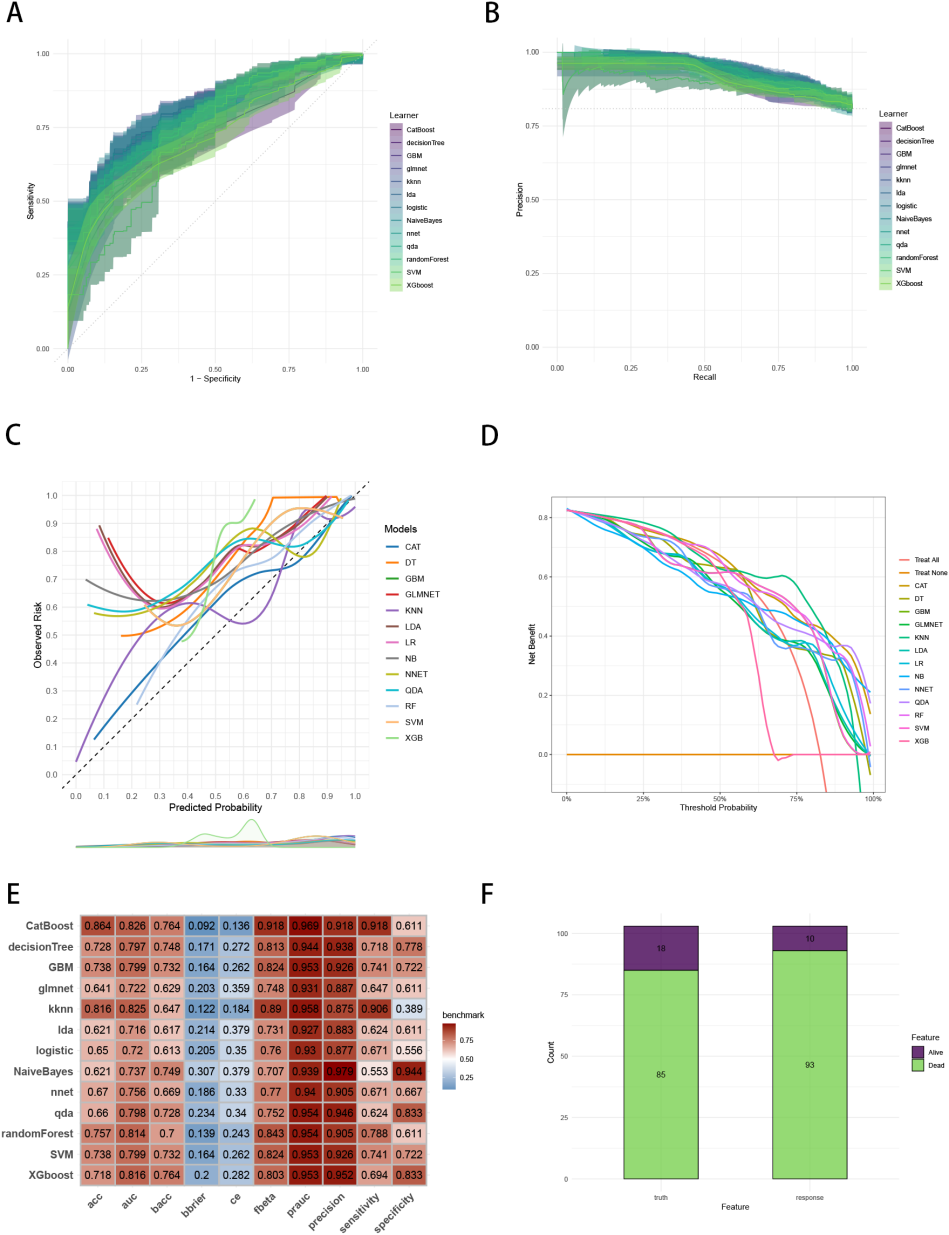
Evaluation of the ML models in the external validation set. **(A)** ROC curves of different ML models in the external validation set. **(B)** PR curves of different ML models in the external validation set. **(C)** Calibration curves of different ML models in the external validation set. **(D)** DCA curves of different ML models in the external validation set. **(E)** The performance of 13 ML models in terms of AUC, PRAUC, accuracy, sensitivity, specificity, precision, cross-entropy, Brier scores and Balanced Accuracy (bacc) and F Beta Score (fbeta) in the external validation set. **(F)** Confusion matrix of the best ML model in the external validation set. ML, machine learning; CAT, categorical boosting; LR, logistic regression; DT, decision tree; RF, random forest; XGB, extreme gradient boosting; GBM, gradient boosting machine; NB, Naive Bayes; LDA, linear discriminant analysis; QDA, quadratic discriminant analysis; NNET, neural network; GLMNET, generalized linear models with elastic net regularization; SVM, support vector machine; KNN, k-nearest neighbor.

To establish a precise model to predict three-year survival, we utilized the eight variables (“AJCC_Stage”, “Chemotherapy”, “Age”, “Grade”, “Lung_Metastasis”, “M_Stage”, “Surgery_Type”, “Tumor_Size”) selected by RFE and RRA. A total of 13 ML models, comprising CatBoost, RF, SVM, XGB, DT, GBM, KNN, LR, NBC, LDA, QDA, NNET and GLM algorithms, were developed by incorporating the above selection of eight variables in the training set. Hyperparameters were fine-tuned by performing 5-cross validation and random searches. Then we evaluated the 13 ML models in the internal validation and external validation cohorts, respectively. Finally, ROC curves analysis found that CatBoost model had the highest AUC in the training (0.932 [0.924, 0.939]), internal validation (0.899 [0.873, 0.934]) and external validation (0.826 [0.735, 0.919]) cohorts (**Figures 3A**, **4A**, **5A**). CatBoost model has the accuracy of 0.839 [0.831, 0.847], sensitivity of 0.872 [0.858, 0.887], specificity of 0.803 [0.784, 0.825] and precision of 0.832 [0.821, 0.853]. After grid search in hyperparameter tuning, the best hyperparameter metric of CatBoost was depth, 5; learning_rate, 0.01678325; iterations, 548; 12_leaf_reg, 7.409126. The precision-recall curves (PRC) revealed that CatBoost model was powerful in handling imbalanced datasets (**Figures 3B**, **4B**, **5B**). Calibration plots showed that CatBoost algorithm had the best fitting ability and could accurately predict three-year survival (**Figures 3C**, **4C**, **5C**). This indicates that the model’s probability estimates are reliable and well-calibrated, as it ensures that the risk estimates provided by the model can be trusted to reflect the true likelihood of patient outcomes. DCA curves suggested that CatBoost algorithm had the best clinical application value and could effectively help predict three-year survival (**Figures 3D**, **4D**, **5D**). This implies that using the CatBoost model to guide clinical decision-making would result in more effective identification of patients who are likely to benefit from certain interventions, such as more aggressive treatment or intensive monitoring. The accuracy, sensitivity, specificity, precision, cross-entropy, Brier scores, Balanced Accuracy (bacc) and F Beta Score (fbeta) of the 13 ML models were calculated to comprehensively evaluate the model performance, which revealed that CatBoost model was robust in predicting three-year survival (**Figures 3E**, **4E**, **5E**). The results of tenfold cross-validation indicated that CatBoost exhibited the best performance (**Figure 3F**). Confusion matrix displayed the outstanding predictive ability of CatBoost in the internal validation and external validation cohorts (**Figures 4F**, **5F**). Therefore, CatBoost was chosen as the best model for the next step.

### Model interpretation

**Figure 6:**
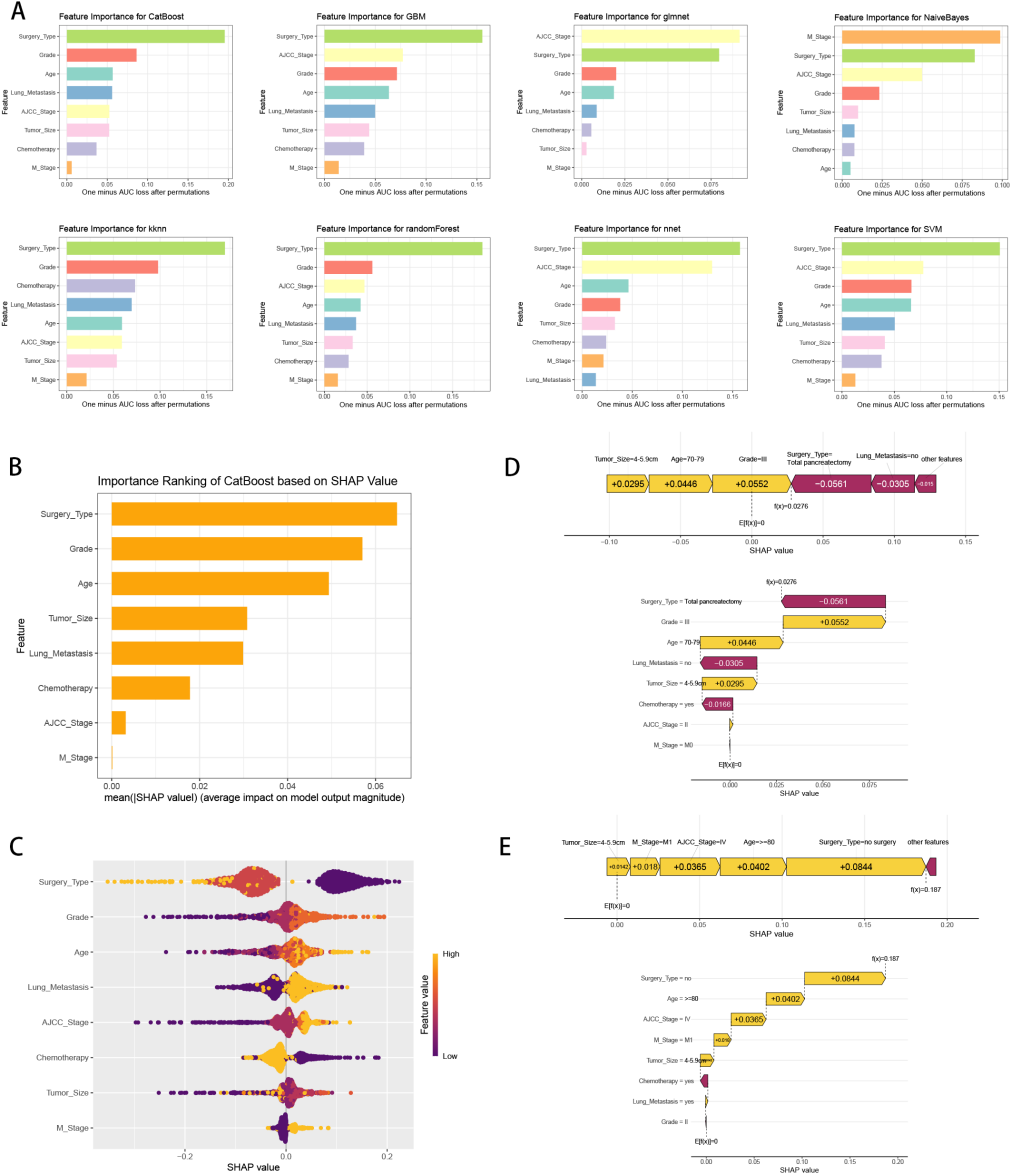
ML model interpretation. **(A)** Importance ranking of features in eight ML prediction algorithms (CatBoost, GBM, GLM, NB, KNN, RF, NNET and SVM). **(B)** The importance ranking of different variables according to the mean (|SHAP value|) using the optimal CatBoost model. **(C)** The importance ranking of different risk factors with stability and interpretation using the optimal CatBoost model. The higher SHAP value of a feature is given, the higher risk of distant metastasis the patient would have. The yellow part in feature value represents higher value. **(D)** SHAP value explanation in a classical sample with three-year survival. **(E)** SHAP value explanation in a classical sample without three-year survival.

We calculated the feature importance rankings of each ML models and illustrated eight of them, including CatBoost, GBM, GLM, NB, KNN, RF, NNET and SVM (**Figure 6A**). The importance scores were determined by leveraging the inherent attributes of various ML algorithms, which revealed that the risk factors most associated with three-year survival were “Surgery Type”, “AJCC Stage” and “M Stage”. Subsequently, we utilized SHAP framework to interpret CatBoost model. We illustrated all of the risk factors evaluated by the mean absolute SHAP value, which revealed that “Surgery Type” was the most impactful variable (**Figure 6B**). Besides, beeswarm plot elucidated the influence of various risk factors on three-year survival (**Figure 6C**). The y-axis denotes the magnitude of the risk factors, while the x-axis represents their impact on the model’s output, specifically three-year survival, as measured by the SHAP value. It was observed that no surgery, higher grade, older age, have lung metastasis, no chemotherapy, higher AJCC stage and M1 stage are associated with an increased likelihood of death in three-year follow-up. To illustrate the model’s interpretability, we highlighted two representative cases. SHAP values were used to understand the impact of each feature on the model’s prediction. In our study, lower SHAP values indicate a higher likelihood of three-year survival, while higher SHAP values suggest a higher probability of death within the three-year follow-up. We chose median value (0.0962) as the cut-off point for predicting the low or high probability of three-year survival. For instance, the first patient with three-year survival had a lower SHAP value and a prediction score of 0.0276, indicating a higher likelihood of three-year survival (**Figure 6D**). In contrast, the second patient without three-year survival showed a higher SHAP value and a prediction score of 0.187, suggesting a higher probability of death in three-year follow-up (**Figure 6E**).

### Prognostic model establishment and performance

**Figure 7:**
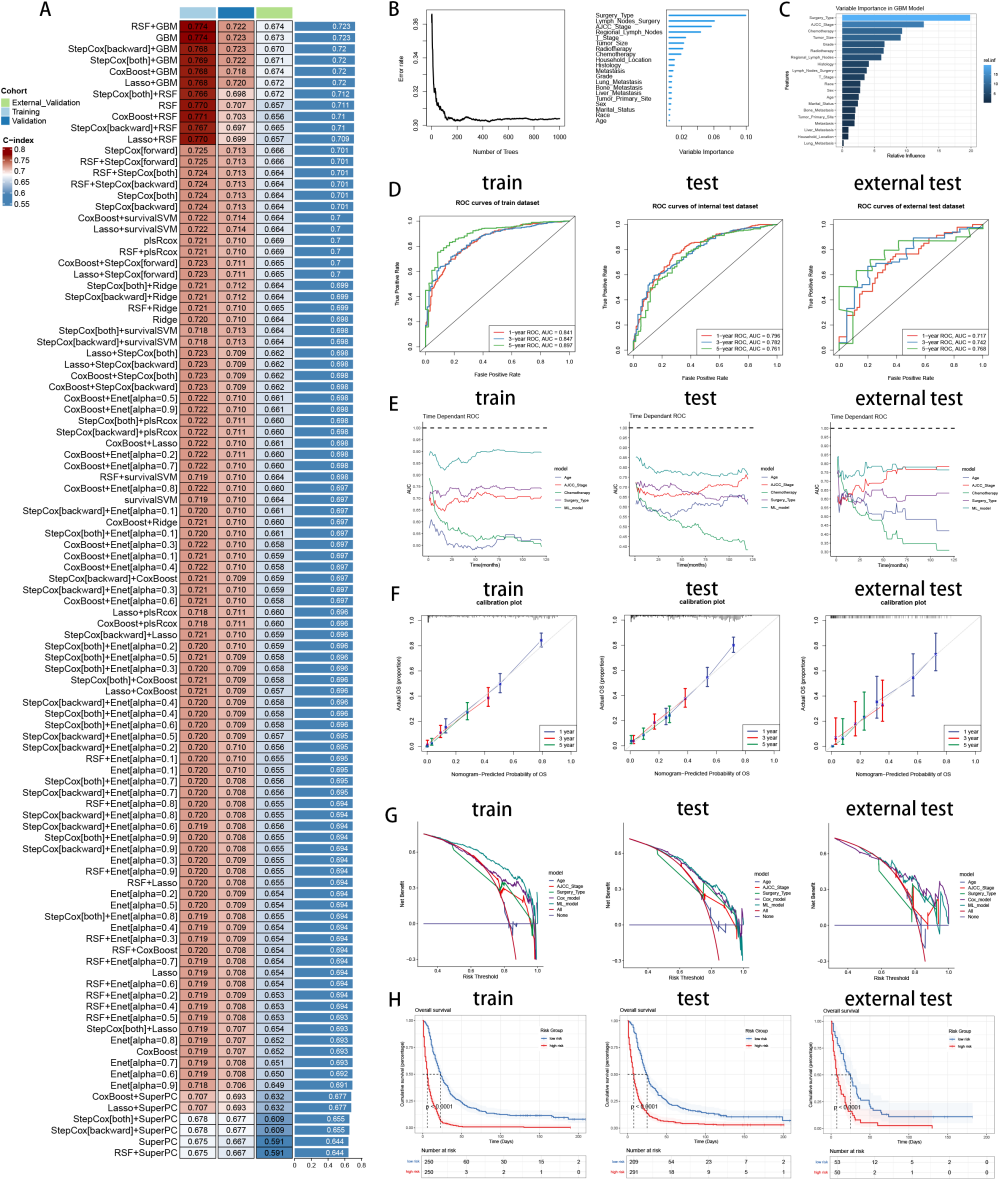
Establishment and validation of prognostic model for pancreatic cancer patients. **(A)** A total of 101 kinds of prognostic models via a leave-one-out cross-validation framework and further calculated the C-index of each model. **(B)** Feature selection process by RSF algorithm. **(C)** Model construction by GBM algorithm and visualization of feature importance. **(D)** ROC curves of ML model in training, internal validation and external validation cohorts. **(E)** Time dependent AUC values of ML model in training, internal validation and external validation cohorts. **(F)** Calibration curves of ML model in training, internal validation and external validation cohorts. **(G)** DCA curves of ML model in training, internal validation and external validation cohorts. **(H)** K-M curves of low-risk and high-risk groups divided by ML model in training, internal validation and external validation cohorts. Left: training cohort, Middle: internal validation cohort, Right: external validation cohort.

To explore the prognostic values of multiple variables, we performed univariate and multivariate Cox analysis to found that “Sex” (HR 0.934(0.907-0.963)), “Race” (HR 1.071(1.022-1.123)), “Age” (HR 1.19(1.119-1.264)), “Marital_Status” (HR 1.137(1.086-1.191)), “Household_Income” (HR 0.866(0.837-0.896)), “Household_Location” (HR 0.945(0.899-0.993)), “Tumor_Primary_Site” (HR 0.946(0.91-0.983)), “Histology” (HR 0.706(0.66-0.755)), “Grade” (HR 1.334(1.271-1.401)), “Tumor_Size” (HR 1.325(1.238-1.417)), “AJCC_Stage” (HR 0.712(0.643-0.789)), “T_Stage” (HR 1.127(1.024-1.242)), “Surgery_Type” (HR 0.491(0.45-0.535)), “Lymph_Nodes_Surgery” (HR 0.846(0.761-0.94)), “Regional_Lymph_Nodes” (HR 0.709(0.65-0.774)), “Radiotherapy” (HR 0.905(0.873-0.938)), “Chemotherapy” (HR 0.582(0.562-0.602)), “Bone_Metastasis” (HR 1.196(1.006-1.422)), “Liver_Metastasis” (HR 1.338(1.224-1.463)), “Lung_Metastasis” (HR 1.338(1.224-1.463)) and “Metastasis” (HR 0.781(0.709-0.861)) were independent prognosis variables for predicting OS in pancreatic cancer patients (P < 0.05, **Table 3**). Incorporating these clinical variables, 101 prognostic ML algorithm combinations were constructed via LOOCV framework. The C-index of each ML combination was calculated in training, internal validation and external validation datasets (**Figure 7A**). Among top five ML combinations with highest C-index across three cohorts, logarithmic loss, recall and decision calibration were calculated to assess the model performances, discovering the well calibration and precision of “RSF+GBM” model (**Supplementary Figure 1**). The best ML model combination was “RSF+GBM”, which was established based on RSF algorithm in feature selection (**Figure 7B**), and GBM algorithm in model construction (**Figure 7C**), with the highest average C-index (0.723) across three datasets (**Figure 7A**). Finally, a 20-variable “RSF+GBM” prognostic ML model was accordingly established to predict OS of pancreatic patients, with “Surgery Type” being the most significant variable both in the feature importance visualization of RSF and GBM model (**Figures 7B, C**). ROC curves of 1-, 3- and 5-year OS showed well specificity of “RSF+GBM” model (**Figure 7D**). Time dependent ROC curves indicated that the curve of “RSF+GBM” model was upper than other curves at most of the time points, indicating that “RSF+GBM” model remarkably outperformed conventional clinical variables in capability of discrimination and prediction (**Figure 7E**). Calibration curves (**Figure 7F**) and DCA curves (**Figure 7G**) showed that “RSF+GBM” model is well-behaved in accuracy and clinical benefit. Based on risk scores calculated by GBM algorithm, we utilized the median risk score to divide patients in the training, internal validation and external validation cohorts into low-risk and high-risk groups, respectively. Obliviously, the low-risk group owned a relatively longer OS than the high-risk group in the training, internal validation and external validation cohorts, respectively (**Figure 7H**). The K-M curves validated the capability of risk stratification of “RSF+GBM” model. All these metrics collectively indicated that “RSF+GBM” model demonstrated stability and robustness in model performances. In conclusion, we have successfully developed a “RSF+GBM” model to predict OS in pancreatic cancer patients, which outperforming other models and was well behaved in model performances.

**Table 3:** Univariate and multivariate cox regression analysis of pancreatic cancer patients for overall survival in the training cohort.

Variable	Univariable cox analysis		Multivariate cox analysis	
term	HR (95%CI)	p.value	HR (95%CI)	p.value
Sex: Male	Reference			
Female	0.96 (0.94-0.99)	0.012	0.934 (0.907-0.963)	<0.001
Age: <50	Reference			
50-59	1.09 (1.02-1.16)	0.008	1.114 (1.045-1.188)	0.001
60-69	1.12 (1.06-1.19)	<0.001	1.19 (1.119-1.264)	<0.001
70-79	1.33 (1.25-1.41)	<0.001	1.412 (1.326-1.502)	<0.001
>=80	1.76 (1.64-1.88)	<0.001	1.598 (1.487-1.717)	<0.001
Race: White	Reference			
Black	1.12 (1.07-1.18)	<0.001	1.071 (1.022-1.123)	0.004
Other	0.96 (0.91-1.01)	0.121	1.022 (0.97-1.077)	0.41
Marital_Status: Married	Reference			
Unmarried	1.11 (1.06-1.16)	<0.001	1.137 (1.086-1.191)	<0.001
Widowed or divorced	1.23 (1.19-1.27)	0	1.162 (1.121-1.206)	<0.001
Unknown	1.07 (0.98-1.16)	0.151	1.07 (0.981-1.167)	0.126
Household_Income: <$70,000	Reference			
>=$70,000	0.89 (0.87-0.92)	<0.001	0.866 (0.837-0.896)	<0.001
Household_Location: Rural				
Urban	0.91 (0.87-0.95)	<0.001	0.945 (0.899-0.993)	0.025
Tumor_Primary_Site: Pancreas Head	Reference			
Pancreas Body or Tail	1.17 (1.13-1.21)	<0.001	0.946 (0.91-0.983)	0.005
Other	1.3 (1.25-1.36)	<0.001	1.004 (0.96-1.051)	0.858
Histology: Adenomas and adenocarcinomas	Reference			
Ductal and lobular neoplasms	0.71 (0.68-0.73)	<0.001	1.022 (0.986-1.059)	0.236
Cystic, mucinous and serous neoplasms	0.59 (0.55-0.63)	0	0.706 (0.66-0.755)	<0.001
Other	0.89 (0.79-1)	0.05	0.876 (0.776-0.988)	0.032
Grade: Well differentiated I	Reference			
Moderately differentiated II	1.25 (1.2-1.32)	<0.001	1.334 (1.271-1.401)	<0.001
Poorly differentiated III	1.74 (1.66-1.83)	<0.001	1.718 (1.635-1.806)	<0.001
Undifferentiated anaplastic IV	1.74 (1.53-1.99)	<0.001	1.485 (1.302-1.694)	<0.001
Summary_Stage: Localized	Reference			
Regional	1.62 (1.53-1.71)	<0.001	1.847 (1.641-2.079)	<0.001
Distant	3.78 (3.56-4)	<0.001	2.136 (1.935-2.357)	<0.001
AJCC_Stage: I	Reference			
II	1.56 (1.48-1.65)	<0.001	0.712 (0.643-0.789)	<0.001
III	2.76 (2.58-2.95)	<0.001	0.762 (0.687-0.846)	<0.001
IV	4.59 (4.32-4.88)	<0.001	NA (NA-NA)	NA
T_Stage: T1	Reference			
T2	2 (1.84-2.17)	<0.001	1.066 (0.969-1.172)	0.19
T3	1.85 (1.72-2)	<0.001	1.127 (1.024-1.242)	0.015
T4	3.4 (3.13-3.69)	<0.001	1.15 (1.029-1.285)	0.014
N_Stage: N0	Reference			
N1	1.05 (1.02-1.08)	0.002	1.036 (0.988-1.087)	0.148
M_Stage: M0	Reference			
M1	1.96 (1.9-2.02)	<0.001	1.017 (0.962-1.075)	0.553
Tumor_Size: <2cm	Reference			
2-3.9cm	1.6 (1.51-1.7)	<0.001	1.325 (1.238-1.417)	<0.001
4-5.9cm	2.12 (1.99-2.25)	<0.001	1.514 (1.41-1.625)	<0.001
6-7.9cm	2.46 (2.28-2.65)	<0.001	1.722 (1.581-1.875)	<0.001
>8cm	2.22 (2.02-2.44)	<0.001	1.629 (1.467-1.808)	<0.001
Unknown	3.01 (2.78-3.26)	<0.001	1.439 (1.318-1.572)	<0.001
Surgery_Type: No Surgery	Reference			
Local or partial pancreatectomy	0.31 (0.3-0.32)	<0.001	0.491 (0.45-0.535)	<0.001
Total pancreatectomy	0.33 (0.31-0.34)	<0.001	0.507 (0.462-0.556)	<0.001
Lymph_Nodes_Surgery: No or biopsy only	Reference			
1-3 regional lymph nodes removed	0.41 (0.38-0.44)	<0.001	0.991 (0.888-1.105)	0.868
4 or more regional lymph nodes removed	0.33 (0.32-0.35)	<0.001	0.846 (0.761-0.94)	0.002
Regional_Lymph_Nodes: No nodes were examined	Reference			
Negative	0.25 (0.24-0.27)	<0.001	0.709 (0.65-0.774)	<0.001
Positive	0.41 (0.39-0.42)	<0.001	1.03 (0.941-1.128)	0.524
Unknown	0.71 (0.53-0.94)	0.018	0.914 (0.682-1.225)	0.547
Chemotherapy: None/Unknown	Reference			
Yes	0.65 (0.63-0.67)	<0.001	0.582 (0.562-0.602)	<0.001
Radiotherapy: None/Unknown	Reference			
Yes	0.69 (0.67-0.71)	<0.001	0.905 (0.873-0.938)	<0.001
Metastasis: No	Reference			
Yes	1.58 (1.54-1.63)	<0.001	0.781 (0.709-0.861)	<0.001
Bone_Metastasis: No	Reference			
Yes	2.82 (2.39-3.33)	<0.001	1.196 (1.006-1.422)	0.042
Unknown	1.19 (1.16-1.23)	<0.001	1.186 (0.636-2.21)	0.591
Brain_Metastasis: No	Reference			
Yes	5.37 (2.23-12.9)	<0.001	1.822 (0.747-4.446)	0.187
Unknown	1.18 (1.15-1.22)	<0.001	0.909 (0.498-1.659)	0.756
Liver_Metastasis: No	Reference			
Yes	2.99 (2.84-3.15)	<0.001	1.338 (1.224-1.463)	<0.001
Unknown	1.34 (1.3-1.39)	<0.001	1.753 (1.27-2.419)	0.001
Lung_Metastasis: No	Reference			
Yes	2.77 (2.52-3.04)	<0.001	1.338 (1.224-1.463)	<0.001
Unknown	1.22 (1.19-1.26)	<0.001	1.753 (1.27-2.419)	0.001

## Discussion

Pancreatic cancer is among the most invasive and deadly malignancies, with projections suggesting it could become the second leading cause of cancer-related deaths by 2030 (21). Although radical surgery offers a chance for a cure, high rates of postoperative recurrence and mortality remain a significant concern (22). Given these challenges, accurately predicting survival rates and identifying prognostic risk factors is of critical importance for pancreatic cancer patients. In this study, we focused on developing novel predictive and prognostic ML models to early predict three-year survival, and to forecast the prognosis of pancreatic cancer patients. By gathering clinical data on several key variables and establishing ML models via benchmark framework, we were able to calculate risk scores related to prediction and prognosis, enabling us to precisely predict the probability of three-year survival and the prognosis of patients. The model analyzes various clinical and demographic features to provide a risk score for three-year survival and prognosis, which helps clinicians determine the intensity and type of treatment required for each patient, outperforming the existing models without ML algorithms (23, 24).

The clinical importance of this work lies in its potential to enhance patient management and treatment planning for those diagnosed with pancreatic cancer. By providing an accurate risk stratification tool, our model can significantly aid clinicians in making more informed, personalized treatment decisions. For instance, patients identified as high-risk for three-year mortality could be prioritized for aggressive surgical interventions, adjuvant therapies, and closer post-operative monitoring, which may improve their chances of survival. Conversely, patients deemed low-risk could benefit from less intensive treatments, thereby avoiding the potential side effects and complications associated with overtreatment. Additionally, the model’s predictions can help the selection of adjuvant therapies, the frequency of follow-up visits, and the need for additional laboratory tests. By integrating the prediction model into clinical workflows, we enable data-driven decision-making that optimizes patient outcomes and resource allocation. As a result, it helps in standardizing care across different healthcare providers and institutions, potentially reducing variability in treatment approaches and outcomes for pancreatic cancer patients.

Moreover, the highlight of our study lies in showcasing how interpretable ML algorithms, particularly through the use of SHAP values, can effectively decipher key factors contributing to predict three-year survival. CatBoost algorithm is a gradient boosting framework based on the symmetric decision tree (oblivious trees) algorithm, which boasts high accuracy and requires fewer parameters, making it efficient and effective in handling categorical features (25). CatBoost’s performance rivals that of other advanced machine learning algorithms, demonstrating its superiority in many applications. But the black-box feature of CatBoost model necessitated its interpretation and explanation with vivid figures. CatBoost’s SHAP summary plots and force maps serve as valuable tools, offering clinicians a visual and intuitive means to understand and identify the critical features influencing three-year survival, which not only elucidates the pivotal risk factors but also improves the interpretability of ML models in clinical settings. Meanwhile, several advanced ML techniques, including feature selection through RFECV, hyperparameter optimization with GridSearchCV, and addressing sample imbalance using SMOTE oversampling, had significantly enhanced the prediction accuracy for the probability of three-year survival. Overall, our precise ML prediction model allowed clinicians to schedule personalized treatment plans, helping them tailor therapy methods in time and enhance prognosis of pancreatic cancer patients.

Researchers have previously shown that old age, high histological grade, large tumor size, AJCC stage, surgery type and metastasis are associated with poorer long-term survival outcomes for pancreatic cancer patients (26, 27). In clinical practice, serum CA199 and CEA levels are commonly used biomarkers in pancreatic cancer, and high levels of CA199 are generally associated with a worse prognosis. Meanwhile, the methylation status of NPTX2, BMP3 and SPARC genes plays an important role in the prognosis of pancreatic cancer. Researchers suggest that methylation of these genes could be used as non-invasive biomarkers to assess prognosis and monitor disease progression in patients with pancreatic cancer (28). In our analysis, we performed univariate and multivariate logistic and cox regression analyses to discover important predictive factors for three-year survival, as well as independent risk factors for prognosis. Based on clinical variables which can be easily obtained during clinical practices, we succeeded in constructing a powerful CatBoost model to early predict three-year survival.

In our research, we observed that patients with pancreatic cancer who undergo surgical resection demonstrated significantly improved survival rates, as supported by Hester et al.’s analysis of the National Cancer Database (29). However, surgery alone is often insufficient for achieving long-term survival, with median survival times typically ranging between 8 to 10 months, frequently accompanied by tumor recurrence (30). Chemotherapy, both as a neoadjuvant (preoperative) and adjuvant (postoperative) treatment, has been identified through logistic and cox regression analyses as a key independent factor in enhancing patient survival. Specifically, adjuvant chemotherapy has been shown to double median survival rates compared to patients who do not receive it, while neoadjuvant chemotherapy improves overall survival and increases the likelihood of R0 resection, making it a valuable treatment option (31). Additionally, age is an independent risk factor, with older patients exhibiting lower survival rates, likely due to diminished immunity and physical decline, which is also common in other types of cancer. Moreover, we found that race does play a role in pancreatic cancer prognosis. African Americans have a higher rate of pancreatic cancer than other racial groups, and their overall survival rate is lower. This difference may be related to a variety of factors, including socioeconomic status, access to and quality of health care, and genetic and environmental factors (32).

Gender can influence the prognosis of pancreatic cancer, though the impact is complex and varies depending on several factors (33). Our analysis results show that women generally have a slightly better overall survival (OS) compared to men. This improved survival in women has also been observed in studies analyzing the outcomes of both standard treatments and more aggressive chemotherapy regimens like FOLFIRINOX (34). Moreover, our analysis displayed that metastasis in pancreatic cancer significantly affected prognosis, with different metastatic sites influencing survival outcomes differently (35). Common sites of distant metastasis in pancreatic cancer include the peritoneum and liver, followed by the lungs, bones, and other organs (36). Liver metastasis is the most common and is associated with the poorest prognosis, often due to the liver’s role in filtering blood and its involvement in the metabolism of cancer drugs. Lung metastasis, while also serious and crucial, generally presents a slightly better prognosis compared to liver involvement. Peritoneal metastasis reflects a more extensive spread of the disease within the abdominal cavity. This type of metastasis is particularly challenging because it often leads to complications such as ascites (the accumulation of fluid in the abdomen), which can be difficult to manage and severely impacts the patient’s quality of life. Overall, the presence of metastasis generally indicates an advanced disease and a poor prognosis, due to the difficulty of achieving complete surgical resection and the challenges in effectively targeting metastatic sites with systemic therapies.

While this study boasts certain strengths, it also faces multiple limitations. Firstly, we calculated the needed sample size for our external validation set, but we were unable to gather a large enough external validation set due to the limited number of patients with complete follow-up information. Although we recognize that large sample sizes improve the reliability of model evaluations, we have tried to collect the largest sample size available in the current research environment. Despite the small set of external validations, we maximize the reliability of validation by using a 10-fold cross-validation approach to assess the model’s ability to generalize. In future studies, we plan to increase the sample size of the external validation set, thereby further verifying the universality and reliability of the model. Secondly, our study relies on retrospective datasets sourced from the SEER database, causing possibility of selection bias. Meanwhile, the inconsistent data collection across multiple hospitals, as well as the retrospective study design, led to some missing clinical feature data. Thirdly, the absence of some key clinicopathological parameters is noted, due to the unavailability of image data and laboratory test indicators from the SEER database. The study predominantly utilizes baseline characteristics and routine clinical data as variables, without some important indicators such as CA199, CEA and KRAS gene mutation. To enhance the model’s predictive accuracy and identify risk factors, a broad range of features was included, which somewhat complicates its practical application in a clinical setting. Finally, the model has yet to be implemented in clinical practice, thus necessitating prospective, multicenter, and large-scale validations to fully ascertain its generalizability in the future.

## Conclusions

In this study, we developed a CatBoost predictive model based on ML benchmark framework, to more accurately predict three-year survival for pancreatic cancer patients, surpassing traditional models in effectiveness and performances. We successfully identified significant predictive factors for three-year survival of pancreatic cancer. Meanwhile, we establish a GBM prognostic model to predict prognosis of pancreatic cancer patients for achieving personalized medicine. This research laid a foundation for future efforts aimed at enhancing three-year survival prediction and prognosis forecasting, which could help clinicians in decision making and therapy plan tailoring.

## Data Availability

The raw data supporting the conclusions of this article will be made available by the authors, without undue reservation.
