# Supplementary material for: Personalized three-year survival prediction and prognosis forecast by interpretable machine learning for pancreatic cancer patients: a population-based study and an external validation
Source: Front Oncol. 2024 Oct 21;14:1488118. doi: 10.3389/fonc.2024.1488118 (PMC11532159; doi:10.3389/fonc.2024.1488118)

Supplementary Figure 1

Logarithmic loss, recall and decision calibration of top 5 prognostic machine learning models in the training, internal validation and external validation sets.
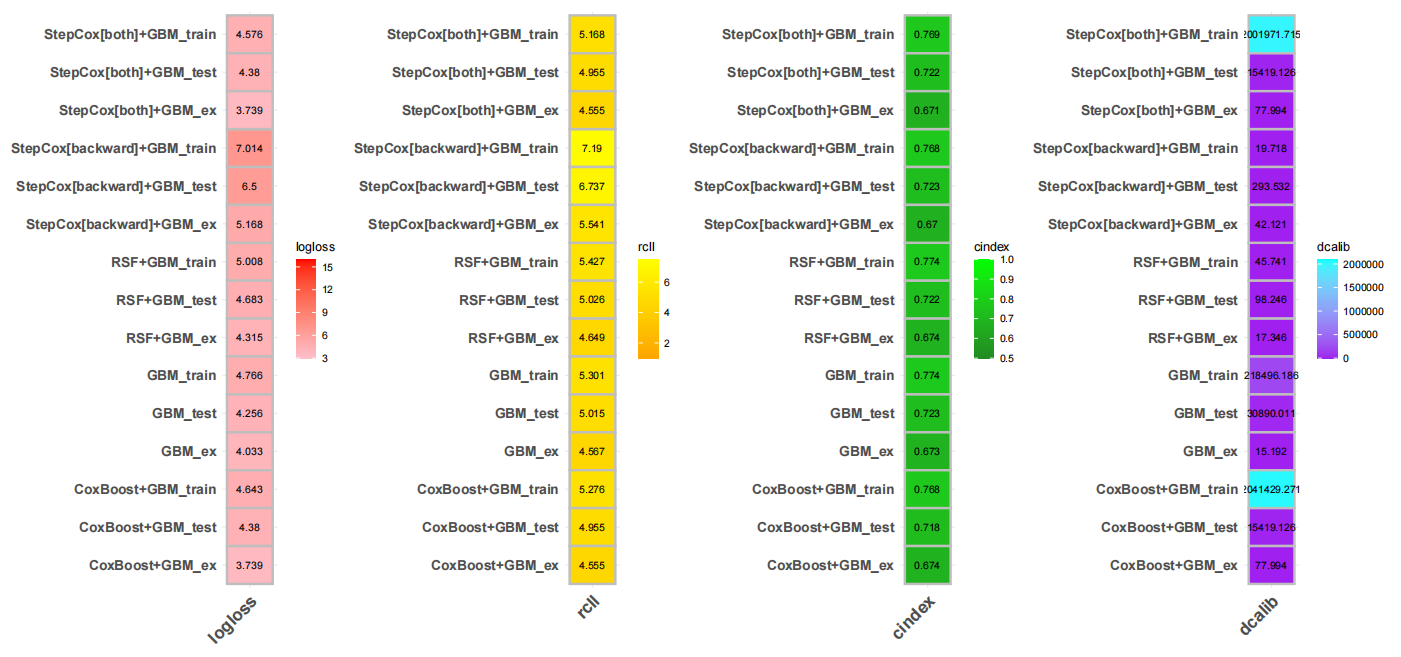

Supplement: Supplementary file 1 [file DataSheet1.docx]
